# Polygenic evidence and overlapped brain functional connectivities for the association between chronic pain and sleep disturbance

**DOI:** 10.1038/s41398-020-00941-z

**Published:** 2020-07-24

**Authors:** Jie Sun, Wei Yan, Xing-Nan Zhang, Xiao Lin, Hui Li, Yi-Miao Gong, Xi-Mei Zhu, Yong-Bo Zheng, Xiang-Yang Guo, Yun-Dong Ma, Zeng-Yi Liu, Lin Liu, Jia-Hong Gao, Michael V. Vitiello, Su-Hua Chang, Xiao-Guang Liu, Lin Lu

**Affiliations:** 1 Center for Pain Medicine, Peking University Third Hospital, Beijing, 100191 China; 2 Peking University Sixth Hospital, Peking University Institute of Mental Health, NHC Key Laboratory of Mental Health (Peking University), National Clinical Research Center for Mental Disorders (Peking University Sixth Hospital), Beijing, 100191 China; 3 Department of Anesthesiology, Peking University Third Hospital, Beijing, 100191 China; 4 Center for MRI Research, Peking University, Beijing, 100871 China; 5 Department of Psychiatry and Behavioral Sciences, University of Washington, Seattle, WA 98195 USA; 6 Research Unit of Diagnosis and Treatment of Mood Cognitive Disorder (2018RU006), Chinese Academy of Medical Sciences, Beijing, 100191 China; 7 Department of Orthopedics, Peking University Third Hospital, Beijing, 100191 China

**Keywords:** Genomics, Neuroscience

## Abstract

Chronic pain and sleep disturbance are highly comorbid disorders, which leads to barriers to treatment and significant healthcare costs. Understanding the underlying genetic and neural mechanisms of the interplay between sleep disturbance and chronic pain is likely to lead to better treatment. In this study, we combined 1206 participants with phenotype data, resting-state functional magnetic resonance imaging (rfMRI) data and genotype data from the Human Connectome Project and two large sample size genome-wide association studies (GWASs) summary data from published studies to identify the genetic and neural bases for the association between pain and sleep disturbance. Pittsburgh sleep quality index (PSQI) score was used for sleep disturbance, pain intensity was measured by Pain Intensity Survey. The result showed chronic pain was significantly correlated with sleep disturbance (*r* = 0.171, *p*-value < 0.001). Their genetic correlation was *r*_g_ = 0.598 using linkage disequilibrium (LD) score regression analysis. Polygenic score (PGS) association analysis showed PGS of chronic pain was significantly associated with sleep and vice versa. Nine shared functional connectivity (FCs) were identified involving prefrontal cortex, temporal cortex, precentral/postcentral cortex, anterior cingulate cortex, fusiform gyrus and hippocampus. All these FCs mediated the effect of sleep disturbance on pain and seven FCs mediated the effect of pain on sleep disturbance. The chronic pain PGS was positively associated with the FC between middle temporal gyrus and hippocampus, which further mediated the effect of chronic pain PGS on PSQI score. Mendelian randomization analysis implied a possible causal relationship from chronic pain to sleep disturbance was stronger than that of sleep disturbance to chronic pain. The results provided genetic and neural evidence for the association between pain and sleep disturbance, which may inform future treatment approaches for comorbid chronic pain states and sleep disturbance.

## Introduction

Sleep disturbance and chronic pain are two common disease states which are bi-directionally interrelated with reciprocal interactions^1,2^. Each of them occurs with great frequency and results in significant public health and socioeconomic burden^3,4^. There is considerable evidence linking pain to disturbed sleep. Sleep disturbance is present in 67–88%^5^ of chronic pain patients, and at least 50%^6^ of individuals with insomnia suffer from chronic pain. It’s crucial to identify overlapped genetic and neural pathways correlated to two symptoms to explore etiological contribution towards the reciprocal relationship.

Genetics has been shown to explain as much as 50% of the variance in pain syndromes^7^ and 25%–45% for insomnia^8^. High genetic correlation (*r*_g_ = 0.69) between pain and sleep disturbance has been reported using twins sample^9^, which implies a possible overlap in the genes affecting both sleep disturbance and chronic pain^10^. Genome-wide association studies (GWAS) have shown that chronic pain was associated with several genes involved in brain function and development and with a range of psychiatric traits^11^. GWAS for sleep disturbance related traits^12^ and insomnia^13,14^ have identified several significant loci and shared genetics with neuropsychiatric and metabolic traits. The large sample size GWAS data provided new ways to assess pleiotropy and genetic correlations between two related traits from a polygenic perspective. Multiple methods have been developed to accomplish this, including linkage disequilibrium (LD) score regression, which uses GWAS summary data to calculate genetic correlations between two traits^15,16^, polygenic score (PGS) association analysis^17^, which uses GWAS summary data of a trait to test whether the effect of a set of single-nucleotide polymorphisms (SNPs) is associated with another phenotype in an independent sample^18^. To our knowledge, the genetic correlation between sleep and pain has not been examined using GWAS data. The genetic correlation between pain and sleep disturbance requires further exploration especially from a polygenic perspective to reveal the mechanisms involved in this relationship.

Genetic association between chronic pain and sleep disturbance makes it especially interesting to search for possible neural mechanisms that may mediate the association. Neuroimaging exploration of the brain mechanisms underlying a bidirectional relationship between sleep disturbance and pain has been dominated by studies mapping the effects of sleep deprivation on pain responsivity^19–23^. Hyperalgesia engendered by sleep loss involves increasing pain reactivity associated with heightened somatosensory cortex activity and a corresponding decrease in insula and striatum activity^22^. Understanding the neural mechanisms underlying the interrelationship of sleep disturbance and chronic pain is a key factor in the development of new therapies, and network-based methods^24–26^ are useful for attaining the goal. Studies have shown that connectivity between the dorsal nexus and dorsolateral prefrontal cortex was susceptible to experimental sleep manipulation^27^. The detection and inhibition of nociceptive inputs could be modulated by the functional dynamics of brain networks^23^. Considering the complex relationship between genetic factors, brain networks and phenotypes, considerable efforts have been made to relate genetic variants to underlying neurobiological aspects using imaging-genetic methods^28^.

One hypothesis is that genetic factors contribute to phenotypes through the mediation of neurobiological changes. Mediation analyses are employed to better understand a known relationship by exploring the underlying mechanism or process by which one variable influences another variable through a mediator variable^29^. Two-sample Mendelian randomization (MR) is widely used to infer causal relationships between two traits based on GWAS summary data^30^ and has been used in many studies^31,32^. These analyses would facilitate to understand the causal relationship between chronic pain and sleep disturbance and the contribution of genetic factors and neurobiological features to this bi-directional relationship.

The goal of this study is to identify the genetic correlation and shared brain abnormalities of modulatory effects of the reciprocal relationships between sleep disturbance and pain, explore the interaction between genetics and aberrant neurobiology from polygenic aspects and inference their causal relationship, which will inform mechanisms involved in this relationship to facilitate new therapies and better treatment.

## Methods

### Participants and data

To explore the relationship between sleep disturbance and chronic pain, we analyzed phenotype data, imaging data and genotype data from 1206 participants (all obtained full informed consent) from the Human Connectome Project (HCP) database (March 2017 public data release) from the Washington University-University of Minnesota (WU-Minn HCP) Consortium. Research procedures and ethical guidelines were followed in accordance with Washington University institutional review board approval. To apply for the genotype data, the study was also approved by the Institutional Review Board of the Peking University Sixth Hospital. In addition, two large sample size GWAS summary data for sleep disturbance and chronic pain were downloaded from published studies for the combined analyses.

### Phenotypes

The sleep phenotype of participants in the HCP was assessed by Pittsburgh Sleep Quality Index (PSQI), a validated self-reported assessment of sleep disturbance^33^. Participants’ self-reported experience of pain was measured by the National Institutes of Health (NIH) Toolbox Pain Intensity Survey^34^. A score was used to measure the average pain intensity in the past 7 days, which was used to measure their chronic pain (CP). More details about the phenotypes are described in the Supplementary Methods and Supplementary Fig. S1. Since the distribution of pain intensity score was not normal, we used a binary variable for pain (chronic pain binary, CPb). According to previous studies^35^, a pain intensity score ≥6 denotes severe pain, which was set as 1, and a pain intensity score ≤5 was set as 0. Because an average pain intensity above 5 leads to disruptions of function and mood, it’s conceptually distinct from mild pain^36,37^.

### Genotype data

The genotype data for the HCP participants was requested from dbGaP under access number phs001364. Among the total 1206 participants, 1142 subjects were genotyped using Infinium Multi-Ethnic Genotyping Array. Quality control for the genotype data was described in Supplementary Methods. Since HCP contained many twins and siblings, after quality control, 429 independent individuals with 1,169,182 SNPs remained for the PGS association analysis. Principal component analysis (PCA) was performed for the genotype of 429 individuals for population stratification. The top 10 principal components (PCs) with a Tracy-Widom test *p*-value < 0.05 (Supplementary Fig. S2) were used as covariates in the subsequent PGS association analyses.

### Imaging data and construction of whole-brain functional network

The HCP participants were scanned on a 3-T connectome-Skyra scanner (Siemens) using standard multiband blood oxygen level dependent (BOLD) acquisition. We acquired the resting-state data preprocessed by the HCP with its uniform method^38^. The participants with four resting-state data (two scans, and two directions for each scan) were used for the construction of whole-brain functional network. After preprocessing, the gray matter of the whole brain was parcellated into 250 regions employing the Shen atlas^39^. Nodal signals were created by averaging the regional blood oxygen level-dependent signals of all voxels within each region. Pearson cross correlations between all pairwise combinations of region signals were calculated for each participant, followed by z transformation to improve normality. The whole-brain functional connectivity network (250 × 250 regions with 31,125 edges) was constructed by further averaging the correlation coefficients of two directions of two scans.

### GWAS summary statistics data of chronic pain and sleep disturbance

Large sample size GWAS summary data on chronic pain and sleep disturbance were used to explore their genetic correlations. The GWAS summary data of chronic pain was from a multisite GWAS of chronic pain in the UK Biobank, which included ~380,000 participants^11^. The PSQI is a widely used measure of sleep quality with high sensitivity (98.7%) and specificity (84.4%)^40^ for identifying primary insomnia^33,41,42^. Because there were no GWAS summary data on sleep disturbance, GWAS summary data on insomnia (i.e., the most common sleep disturbance) from a study of 1,331,010 individuals^13^ was used as a conceptual approximation of sleep disturbance.

### Statistical analysis

**Fig. 1:**
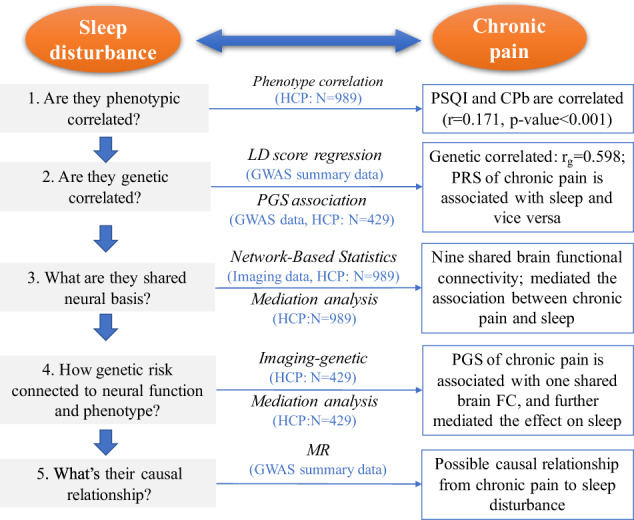
Flowchart of analyses. The analyses that were performed in this study included (1) phenotypic correlation, (2) genetic correlation using LD score regression and polygenic score (PGS) association analysis, (3) shared brain functional connectivity (FC) using network-based statistics method by analyzing rfMRI data, (4) association of the PGS with shared brain functional connectivity, and (5) causal relationship inference using Mendelian randomization (MR). HCP (Human Connectome Project) is the data source. *N* is the sample size for the analysis.

The analyses flowchart is shown in Fig. 1 to explore the relationship between chronic pain and sleep disturbance. All the analyses below used age, gender, race (categorized as white or other), handedness, years of education, body mass index, blood pressure (systolic and diastolic), alcohol abuse diagnosis, smoke history, marijuana dependence diagnosis for lifetime as covariates.

### Phenotypic and genetic correlations between chronic pain and sleep disturbance

The phenotypic correlation between PSQI score and pain CPb was calculated using partial correlation after controlling the covariates as above using SPSS v26. To explore their genetic correlation, LD score regression analysis^15,43^ was conducted by using GWAS summary data on chronic pain^11^ and sleep disturbance^13^. LD score regression has been used in many studies to test the genetic correlations between two traits^16^. Then, we used PGS association analyses^44^ to investigate if the PGS of chronic pain was associated with sleep disturbance and vice versa. The PGS was calculated using GWAS summary data (discovery), and further regressed (linear regression for PSQI, logistic regression for CPb) with phenotype data in the HCP GWAS data (target) (see Supplementary Methods).

### Identifying functional connectivity correlated with pain and sleep disturbance

To address the issue of multiple comparisons during identifying functional connectivity (FC) from the large brain network (31,125 edges), we used the network-based statistic (NBS) method^24^ to identify the FC correlated with PSQI total score. The NBS method is a well-validated tool for brain network association analyses that has been previously been used widely in neuroimaging studies^25,26^. A linear regression model was constructed after controlling for covariates to yield a t-test statistic, then the NBS method identified topological clusters using the test statistic threshold of 3.0 and further computed a family-wise error rate (FWER) corrected p-value for each component using 10,000 permutation testing to address the issue of multiple comparisons. The component with FWER corrected *p*-value < 0.05 was considered significant. The PSQI score associated FCs were further analyzed using logistic regression model for CPb with the same covariates as above to test if the sleep disturbance related FCs were associated with pain. The FCs with FDR adjusted *p*-value < 0.05 was considered significant.

### Association of polygenic score of chronic pain/sleep disturbance with shared FC

To further explore if the shared FCs were associated with the PGS of chronic pain/sleep disturbance, we used PRSice v2^44^ to run the PGS association analysis. Multiple PGS were created for chronic pain/sleep disturbance using the SNPs at *p*-value thresholds from 0 to 0.5 increasing by 0.00005. The association of the PGS with the correlation coefficient of each significant pair of FC was examined in linear regression model adjusting the covariates as above. The p-value threshold with the biggest Nagelkerke’s *R*^2^ was considered as the best-fit threshold. Due to the large number of non-independent tests performed, the *p*-value was adjusted by using 10,000 label-swapping permutations and the adjusted *p*-value < 0.05 was considered significant.

### Mediation analysis

To explore how the shared FCs contribute to the association between chronic pain and sleep disturbance, we used model 4 in PROCESS^45^ to construct mediation models to analyze if the shared FC (mediation variable) mediated the association between chronic pain (independent variable/output variable) and sleep disturbance (output variable/independent variable). The same covariates as above were used in the model. *N* = 10,000 was used for bootstrap. Sobel test was further used to show the *p*-value which denotes if the indirect effect was different with zero. In addition, the mediation function of the shared FC for the association between the PGS and phenotype was assessed using the same models.

### Two-sample Mendelian randomization for causal inference

To explore the causal relationship between chronic pain and sleep disturbance, we conducted two-sample MR analyses using the R package of database and analytical platform MR-Base^30^. The SNP p-value threshold for instrument variables was defined as *p*-value < 5.0 × 10^−8^. To avoid bias in the MR estimates due to LD (*r*^*2*^), clumping was applied using the ‘clump_data’ function with an *r*^*2*^ < 0.001. To combine estimates from individual genetic variants, we applied inverse-variance-weighted (IVW) regression to test the causal effect. Then, we used the MR Egger intercept test to test for directional horizontal pleiotropy, and IVW regression test for variant heterogeneity. Additionally, the MR Egger test^46^, weighted median test^47^, and MR-PRESSO^48^ and Contamination mixture^49^ methods were used for sensitivity tests.

## Results

### Phenotype correlation

After quality controls, total of 989 participants with all four resting-state imaging data, sleep data, pain data, and covariates were used for the phenotypic and neuroimaging analysis. Participants ranged from 22 to 37 years (mean = 28.73, standard deviation = 3.70) with 461 males and 523 females. The demographic data for the participants is shown in Table 1. In the regression models with all covariates, gender, race, handedness, education, BMI, alcohol abuse diagnosis and smoke history were associated with PSQI total score; gender, race and education were associated with CPb. After controlling for covariates, the correlation between PSQI total score and CPb values was significant (*r* = 0.171, *p*-value < 0.001).

**Table 1:** Demographic data for the participants and the correlation of each covariates with the phenotype data.

Variable	Statistics in samples	Correlation with PSQI			Correlation with CPb		
		Beta	SE	*p*-value	Beta	SE	*p*-value
*N*	989						
Age [M, SD]	[28.71, 3.71]	−0.040	0.024	0.099	−0.0019	0.0017	0.256
Gender (% male)	46.81%	0.490	0.186	**0.009**	0.0455	0.0132	**0.001**
Race (% white)	75.83%	−0.567	0.202	**0.005**	−0.0373	0.0144	**0.010**
Handedness [M, SD]	[66.64, 43.30]	−0.006	0.002	**0.004**	−8.56 × 10^−5^	1.40 × 10^−4^	0.541
Years of education [M, SD]	[14.97, 1.76]	−0.140	0.051	**0.006**	−0.0118	0.0036	**0.001**
BMI [M, SD]	[26.27, 4.94]	0.068	0.019	**3.48×10**^−4^	0.0022	0.0013	0.105
BP-systolic [M, SD]	[123.42, 13.80]	−0.011	0.009	0.227	8.36 × 10^−4^	6.41 × 10^−4^	0.192
BP-diastolic [M, SD]	[76.36, 10.56]	0.009	0.011	0.416	5.22 × 10^−4^	7.89 × 10^−4^	0.508
Alcohol abuse diagnosis (% Yes)	14.96%	0.176	0.063	**0.005**	1.27 × 10^−4^	0.0045	0.977
Smoke history (%Yes)	46.11%	0.223	0.072	**0.002**	0.0081	0.0052	0.117
Marijuana dependence diagnosis (%Yes)	9.50%	−0.045	0.317	0.887	0.0027	0.0225	0.906
PSQI score [M, SD]	[4.72, 2.74]	—	—	—	—	—	—
CPb (% Yes)	3.84%	—	—	—	—	—	—

*M* mean, *SD* standard deviation; Yes denotes variable = 1; CPb is the binary chronic pain variable, which was defined as 1 with a pain intensity score ≥ 6, and 0 with a pain intensity score ≤ 5; bold value denotes *p* < 0.05.

### Genetic correlation between pain and sleep disturbance

LD score regression analysis showed chronic pain and sleep disturbance was significantly correlated at the genetic risk (*r*_g_ = 0.5975 (0.0215), *Z*-score = 27.811, *p*-value = 3.20 × 10^−170^). PGS association analysis result (Supplementary Table 1) showed that PGS of chronic pain was significantly associated with PSQI total score (*R*^2^ = 0.0159, *p*-value = 6.47 × 10^−3^, *P*_adjust_ = 0.0324, coefficient=1051.62, SE = 384.29); PGS of sleep disturbance was also significantly associated with CPb (*R*^2^ = 0.1102, *p*-value = 9.50 × 10^−4^, *P*_adjust_ = 4.75 × 10^−3^, coefficient = 24399.30, SE = 7382.47).

### Shared brain FC associated with chronic pain and sleep disturbance

**Fig. 2:**
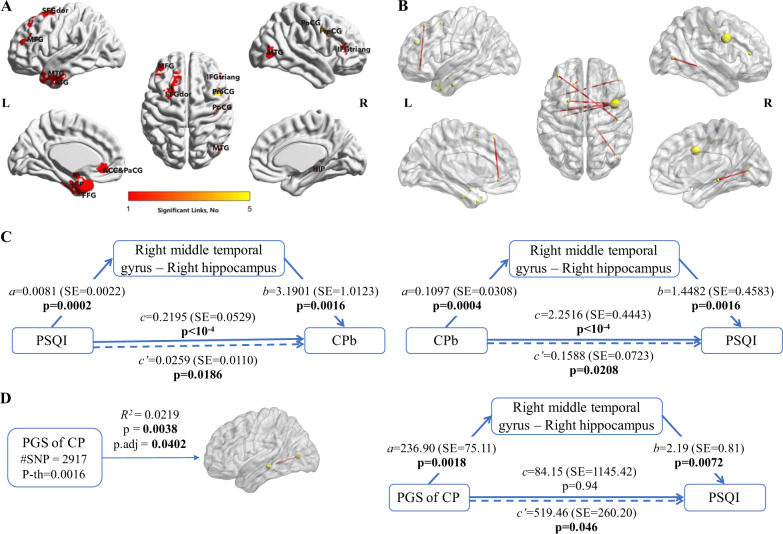
Chronic pain and sleep disturbance shared functional connectivities (FC) and the mediation function of the FC on the association between two phenotypes and the association between polygenic score and phenotypes. **a** Area with the nine FCs shared by PSQI and CPb, color is proportional to the links connected with the node. **b** Nine significant FCs with nodes and edges indicated, node size is proportional to the links connected with the node. **c** Mediation model for the mediation of the FC (“right middle temporal gyrus” - “right hippocampus”) for the correlation between PSQI and CPb. The mediation model for eight other significant FCs are shown in Supplementary Table 2. **d** Association results of PGS of chronic pain with FC (“right middle temporal gyrus” - “right hippocampus”) and mediation model for the indirect effect of PGS of chronic pain (CP) on PSQI through FC (“right middle temporal gyrus” - “right hippocampus”). SFGdor: superior frontal gyrus, dorsolateral; MFG: middle frontal gyrus; MTG: middle temporal gyrus; ITG: inferior temporal gyrus; ACC&PaCG: anterior cingulate & paracingulate gyri; HIP: hippocampus; FFG: fusiform gyrus; IFGtriang: interior frontal gyrus, triangular part; PreCG: precentral gyrus; PoCG: postcentral gyrus. For mediation model, X is independent variable, Y is outcome variable, M is mediation variable. Path *a* is the effect of X on M, path *b* is the effect of M on Y, path *c* is the effect of X on Y (total effect), *c’* is the indirect effect of X on Y. PSQI: Pittsburgh sleep quality index; CP: chronic pain; CPb: chronic pain binary; PGS: polygenic score; #SNP: number of SNPs used to calculate PGS; P-th: p-value threshold to define SNPs with P < P-th to calculate the PGS.

The NBS method obtained one significant network component for PSQI (*p*-value = 0.0288), which included 239 edges and 144 nodes. Further regression for these 239 FCs with CPb showed nine FCs were significantly associated with pain. These nine shared FCs involved the prefrontal cortex (PFC), temporal gyrus, precentral/postcentral gyrus, anterior cingulate gyri, hippocampus and fusiform gyrus (Fig. 2a, b). All of them were significantly correlated with PSQI and CPb (Table 2).

**Table 2:** Shared functional connectivities (FC) between PSQI and CPb. PSQI is the Pittsburgh sleep quality index, CPb is the chronic pain binary.

Brain region 1	Brain region 2	NBS result for PSQI	Correlation between the FC and PSQI	Correlation between the FC and CPb	Regression with CPb					
		statistics	*r*-value	*p*-value	*r* value	*p*-value	coef	SE	*p*-value	fdr_bh
Right middle temporal gyrus	Right hippocampus	3.0727	0.112	0.000443	0.118	0.000211	3.8968	1.0235	0.00014	0.0170
Right precentral gyrus	Left fusiform gyrus	3.7708	0.112	0.000432	0.116	0.0000259	3.2770	0.8615	0.000142	0.0170
Right precentral gyrus	Left hippocampus	3.0871	0.111	0.000469	0.119	0.000172	2.8548	0.7793	0.000249	0.0199
Right precentral gyrus	Left inferior temporal gyrus	3.4775	0.111	0.000467	0.106	0.000825	2.8290	0.8084	0.000466	0.0230
Right inferior frontal gyrus, triangular part	Left temporal pole: middle temporal gyrus	3.2952	0.106	0.000857	0.115	0.000306	2.6520	0.7596	0.000481	0.0230
Right postcentral gyrus	Left middle frontal gyrus	3.3567	0.100	0.001672	0.097	0.002225	2.6683	0.7900	0.000731	0.0275
Right precentral gyrus	Left temporal pole: middle temporal gyrus	3.0579	0.110	0.000551	0.104	0.001047	2.5839	0.7711	0.000805	0.0275
Left superior frontal gyrus, dorsolateral	Left anterior cingulate & paracingulate gyri	3.0846	0.094	0.002955	0.100	0.001624	2.3930	0.7307	0.001057	0.0316
Right precentral gyrus	Left superior frontal gyrus, dorsolateral	3.1769	0.109	0.000598	0.096	0.002542	2.3885	0.7396	0.00124	0.0329

Correlation between the FC and phenotype was from partial correlation analysis after controlling the covariates. Logistic regression model was used for CPb with the same covariates.

Furthermore, we assessed whether the shared FCs mediated the association between PSQI and CPb. The mediation model for the FC “right middle temporal gyrus” - “right hippocampus” is shown in Fig. 2c. The mediation models for the other eight FCs correlated with PSQI and CPb are shown in Supplementary Table 2. Seven out of the nine shared FCs showed mediation function on both the effect of PSQI on CPb and the effect of CPb on PSQI. Connectivity “right precentral gyrus” - “left hippocampus” and “left superior frontal gyrus, dorsolateral” - “left anterior cingulate & paracingulate gyri” mediated the effects of PSQI on CPb but not on the other direction.

### Association of polygenic score of chronic pain/sleep disturbance with brain FC

Given the association of chronic pain and sleep disturbance at both genetic and neural levels, we investigated if the PGS of chronic pain or sleep disturbance was associated with the shared FC, and identified one significant result: the PGS of chronic pain was significantly associated with the FC “right middle temporal gyrus” - “right hippocampus” (permutation adjusted *p*-value = 0.0402) (Fig. 2d). The association of the PGS of sleep disturbance with this FC obtained *p*-value = 9.4 × 10^−3^, but permutation adjusted *p*-value was not significant (adjusted *p*-value = 0.1517) (Supplementary Table 3).

We further analyzed if the PGS, which was associated with the FC (“right middle temporal gyrus” - “right hippocampus”), was associated with the phenotypes (CPb and PSQI) through the mediation function of this FC. The result showed the indirect effect of chronic pain PGS on PSQI mediated through this FC was significant (*p*-value = 0.0459, Fig. 2d), but the indirect effect of sleep disturbance PGS on CPb mediated through this FC was not significant (*p*-value = 0.1233) (Supplementary Table 4).

### Causal relationship between chronic pain and sleep disturbance

The genetic correlation between chronic pain and sleep disturbance may arise from genes with pleiotropic effects. We used a bidirectional, two-sample Mendelian randomization approach to explore the causal relationship between chronic pain and sleep disturbance. As shown in Table 3, putative causal effects of both directions were detected using IVW regression analysis Mendelian randomization test (*p*-value = 1.65 × 10^−14^ for chronic pain as exposure, sleep disturbance as outcome, *p*-value = 9.69 × 10^−7^ for opposite). IVW regression test for variant heterogeneity showed that the heterogeneity was significant for both directions (*p*-value = 2.81 × 10^−4^ and 1.23 × 10^−6^ separately) but the MR Egger tests for directional horizontal pleiotropy were not significant (*p*-value = 0.95 and 0.051 separately). Additional sensitivity tests showed, for chronic pain as exposure, sleep disturbance as outcome, all other four methods had effects in the same direction as the IVW test although p-value for MR Egger test was not significant, but for sleep disturbance as exposure, chronic pain as outcome, weighted median, MR-PRESSO and Contamination mixture had the same direction with IVW test with significance but MR Egger had different direction (Table 3).

**Table 3:** Two-sample MR analysis results for chronic pain and sleep disturbance.

Outcome	#SNP		Mendelian randomization			IVW regression test for variant heterogeneity			MR Egger test for directional horizontal pleiotropy		
			B	SE	*p*-value	Q	*df*	*p*-value	Egger intercept	SE	*p*-value
Sleep disturbance	35	IVW test	0.670	0.087	**1.65×10**^**−14**^	69.879	34	**2.81×10**^**−4**^	−0.00042	0.0066	0.95
		MR Egger	0.696	0.406	0.096						
		Weighted median	0.490	0.097	**3.88×10**^**−7**^						
		MR-PRESSO	0.698	0.092	**6.58×10**^**−9**^						
		Contamination mixture	0.580	0.120	**1.31×10**^**−6**^						
Chronic pain	13	IVW test	0.205	0.042	**9.69×10**^**−7**^	50.322	12	**1.23×10**^**−6**^	0.00944	0.0043	0.051
		MR Egger	−0.030	0.114	0.794						
		Weighted median	0.135	0.038	**3.33×10**^**−4**^						
		MR-PRESSO	0.205	0.042	**3.67×10**^**−4**^						
		Contamination mixture	0.370	0.038	**5.89×10**^**−5**^						

Bold values denote *p* < 0.05.

## Discussion

To our knowledge, this is the first study to examine the underlying genetic and neurologic components of the association between chronic pain and sleep disturbance using a large study sample. Based on the phenotypic correlation between chronic pain and sleep disturbance, which is consistent with earlier findings, we identified their high genetic correlation. At the neural level, we identified shared functional connectivities involving the prefrontal cortex, temporal gyrus, precentral/postcentral gyrus, anterior cingulate gyri, hippocampus and fusiform gyrus, which further mediate the association of chronic pain with sleep disturbance. Furthermore, the chronic pain PGS was associated with the FC “right middle temporal gyrus” - “right hippocampus”, which mediated the association of chronic pain PGS with sleep disturbance. The Mendelian randomization analyses implied a possible causal relationship from chronic pain to sleep disturbance with stronger evidence than the other direction. These findings provide genetic and neural evidence for the co-occurrence of chronic pain and sleep disturbance.

The pleiotropic nature of genes implies that the PGS of chronic pain may also be associated with disturbed sleep and vice versa. The PGS association between chronic pain and sleep disturbance are consistent with phenotypic cross-sectional links and findings from previous twin studies which showed the relationship between sleep and pain to be confounded by shared genetic and environmental factors^9,10,50^.

Genetic components for the development of sleep disturbance and chronic pain likely reflect inherited vulnerability to the development of abnormal functional connectivity. Co-occurrence of sleep disturbance and chronic pain suggests that both might share some common underlying neurophysiological mechanism. Consistent with this possibility, we found that disturbed sleep and chronic pain were significantly correlated with nine FCs involving the prefrontal cortex, temporal gyrus, precentral/postcentral gyrus, anterior cingulate gyri, hippocampus and fusiform gyrus.

Increasing evidence shows that the prefrontal cortex (PFC) is a key brain region correlated with executive function, pain processing and vigilance, awareness, attention^51^ and development of chronic pain. People with higher PSQI have worse performance in tests of working memory and attentional set shifting^52^. Chronic pain patients exhibit significantly less deactivation in medial PFC and abnormal default mode network (DMN) activity while performing a visual attention task^53^. Sleep deprivation research^22,23^ also suggests increased pain sensitivity is positively correlated with FC between executive control network (ECN) and DMN, implies that sleep deprivation impaired cognitive networks could partially contribute to the sleep-pain dyad. The abnormal FC between PFC and anterior cingulate cortex (ACC) we found may demonstrate the deficiency in executive control, thus impairing the ability to transfer from DMN to ECN^54^. Connections of PFC and postcentral gyrus fits the latest model of perception of pain which requires the involvement of PFC^55^. Hyperalgesia might happen through impaired PFC function caused by sleep deficits.

As part of the limbic system, hippocampus could potentiate the sleep disturbance^56^ and make it vulnerable to neuropsychiatric disorders such as depression and chronic pain^57^. Decreased hippocampal neurogenesis is closely associated with memory deficits and aversive affective states in patients with chronic pain^58^. The memory network deficit revealed in chronic pain and sleep disturbed patient might be caused by the aberrant FC between temporal cortex and hippocampus which is demonstrated in our research. We also found the FC between middle temporal gyrus and hippocampus could mediate the association between chronic pain and sleep disturbance. Disrupted hippocampus-PFC connectivity predicts the transition from subacute to chronic pain^59^ and impairment in memory encoding and retrieval, decision-making^60^ and extinction learning^61^.

Furthermore, the shared brain FCs might in charge of properties of chronic pain and sleep disturbance through shared genetic risk, which may lead to sleep disturbances and chronic pain. The association of chronic pan PGS with the FC between right middle temporal gyrus and right hippocampus confirmed this hypothesis. The FC further mediated the indirect effect of the chronic pain PGS on sleep disturbance. Patterns of phenotypic overlap and comorbidity might share a direct etiological links which risk genes confer pleiotropic risk for multiple distinct brain phenotypes^16^.

Pain can be both a cause and a consequence of sleep disturbance. Meta-analysis of longitudinal studies found that a decline in sleep quality and quantity was associated with a two- to three-fold increase in the risk of developing a pain condition^1^. Our research supports both directions of causation, but the causation of sleep disturbance by chronic pain is more strongly supported (all methods had the same effect direction). Longitudinal studies^1,9^ report that pain and sleep often interact and negatively affect each other and the relationship is not unidirectional but more complex. Future longitudinal studies are needed to investigate direction of causation with more ingenious designs^62^.

Finding that chronic pain and sleep disturbance shared neural and genetic underpinnings is significant. Strengths of the present investigation are the large number of participants leading to robust findings, and evidence from both genetic and neural mechanisms obtained by integrating the imaging-genetics results. The mediation and MR causal analyses further helped to uncover the relationship between these two clinically important comorbidities. Currently, pharmacotherapy for chronic pain^63^ and insomnia^64^ has been woefully inadequate. The mediation effect of the aberrant FC that we identified in the present study may indicate potential increased efficacy and reproducibility target of neurostimulation to propose therapies to the patients who suffer from sleep disturbance and complicated by chronic pain.

There were several limitations in our study. The sample in the HCP database is from the young general population. The phenotypic correlation between sleep and pain is weaker than clinical samples, which resulted in relatively few reported pain intensity scores greater than 6. We used a pain intensity score threshold ≥6 to define the binary variable CPb. We also tried to define a threshold ≥3 for the regression analysis of PSQI and FCs, but there was no significant result (data not shown). The logistic regression model with a threshold ≥6 had significant intercept, whereas the threshold ≥3 did not, which may indicate that only severe pain affects brain FC. The PSQI score and pain variable that we used could only represent a portion of sleep disturbance and chronic pain. Additionally, gender is a factor influencing pain and sleep^65,66^. Further research to elucidate the mechanism underlying sex difference in pain and sleep is needed to reduce the disparities. Although the number of participants (*n* = 989) for imaging analysis is relatively large, the sample size for the PGS association analysis was small. Also, causal and mediation relationships between chronic pain and chronic pain results from MR analysis and mediation analyses were analyzed based on the sectional data. The results need further validation and exploration using larger independent samples in studies with longitudinal designs.

## Conclusions

This study explored the association between chronic pain and sleep disturbance from different perspectives. The results revealed their high genetic correlation, identified shared brain functional connectivities mediated their association, built the link from the PGS of chronic pain to brain functional connectivity and further sleep disturbance, and possible causal relationship from chronic pain to sleep disturbance. Our results would have significant implications for understanding the mechanism of sleep and pain comorbidity and imply new treatment and interventions strategy for the comorbidity.

## Supplementary information

Supplementary Information

## References

[1] Afolalu EF, Ramlee F, Tang NKY (2018). Effects of sleep changes on pain-related health outcomes in the general population: a systematic review of longitudinal studies with exploratory meta-analysis. Sleep. Med Rev..

[2] Roy M, Piche M, Chen JI, Peretz I, Rainville P (2009). Cerebral and spinal modulation of pain by emotions. Proc. Natl. Acad. Sci. USA.

[3] Disease GBD, Injury I (2018). Global, regional, and national incidence, prevalence, and years lived with disability for 354 diseases and injuries for 195 countries and territories, 1990-2017: a systematic analysis for the Global Burden of Disease Study 2017. Lancet.

[4] Roenneberg T (2013). Chronobiology: the human sleep project. Nature.

[5] Finan PH, Goodin BR, Smith MT (2013). The association of sleep and pain: an update and a path forward. J. Pain..

[6] Bjurstrom MF, Irwin MR (2016). Polysomnographic characteristics in nonmalignant chronic pain populations: A review of controlled studies. Sleep. Med Rev..

[7] Hocking LJ (2012). Heritability of chronic pain in 2195 extended families. Eur. J. Pain..

[8] Diehl MM (2018). It’s in the genes: a new marker for sex differences in depression and anxiety. Biol. Psychiatry.

[9] Gasperi M, Herbert M, Schur E, Buchwald D, Afari N (2017). Genetic and environmental influences on sleep, pain, and depression symptoms in a community sample of twins. Psychosom. Med.

[10] Pinheiro MB (2018). Genetic and environmental contributions to sleep quality and low back pain: a population-based twin study. Psychosom. Med..

[11] Johnston KJA (2019). Genome-wide association study of multisite chronic pain in UK Biobank. PLoS Genet..

[12] Lane JM (2017). Genome-wide association analyses of sleep disturbance traits identify new loci and highlight shared genetics with neuropsychiatric and metabolic traits. Nat. Genet..

[13] Jansen PR (2019). Genome-wide analysis of insomnia in 1,331,010 individuals identifies new risk loci and functional pathways. Nat. Genet..

[14] Hammerschlag AR (2017). Genome-wide association analysis of insomnia complaints identifies risk genes and genetic overlap with psychiatric and metabolic traits. Nat. Genet..

[15] Bulik-Sullivan BK (2015). LD Score regression distinguishes confounding from polygenicity in genome-wide association studies. Nat. Genet..

[16] Brainstorm, C. et al. Analysis of shared heritability in common disorders of the brain. *Science* **360**, eaap8757 (2018). 10.1126/science.aap8757 PMC6097237 29930110

[17] International Schizophrenia C (2009). Common polygenic variation contributes to risk of schizophrenia and bipolar disorder. Nature.

[18] Clarke TK (2016). Common polygenic risk for autism spectrum disorder (ASD) is associated with cognitive ability in the general population. Mol. Psychiatry.

[19] Seminowicz DA (2019). Pain-related nucleus accumbens function: modulation by reward and sleep disruption. Pain.

[20] Krause AJ (2017). The sleep-deprived human brain. Nat. Rev. Neurosci..

[21] Sardi NF (2018). Chronic sleep restriction increases pain sensitivity over time in a periaqueductal gray and nucleus accumbens dependent manner. Neuropharmacology.

[22] Krause AJ, Prather AA, Wager TD, Lindquist MA, Walker MP (2019). The pain of sleep loss: a brain characterization in humans. J. Neurosci..

[23] Letzen JE (2020). Individual differences in pain sensitivity are associated with cognitive network functional connectivity following one night of experimental sleep disruption. Hum. Brain Mapp..

[24] Zalesky A, Fornito A, Bullmore ET (2010). Network-based statistic: identifying differences in brain networks. Neuroimage.

[25] Zalesky A (2011). Disrupted axonal fiber connectivity in schizophrenia. Biol. Psychiatry.

[26] Cheng W, Rolls ET, Ruan H, Feng J (2018). Functional connectivities in the brain that mediate the association between depressive problems and sleep quality. JAMA Psychiatry.

[27] Bosch OG (2013). Sleep deprivation increases dorsal nexus connectivity to the dorsolateral prefrontal cortex in humans. Proc. Natl. Acad. Sci. USA.

[28] Liu B (2017). Polygenic risk for schizophrenia influences cortical gyrification in 2 independent general populations. Schizophr. Bull..

[29] MacKinnon, D. P. *Introduction to Statistical Mediation Analysis*. (Erlbaum, 2008).

[30] Hemani, G. et al. The MR-Base platform supports systematic causal inference across the human phenome. *Elife* **7**, e34408 (2018). 10.7554/eLife.34408 PMC5976434 29846171

[31] Major Depressive Disorder Working Group of the Psychiatric Genomics, C. (2018). Genome-wide association analyses identify 44 risk variants and refine the genetic architecture of major depression. Nat. Genet..

[32] Vaucher J (2018). Cannabis use and risk of schizophrenia: a Mendelian randomization study. Mol. Psychiatry.

[33] Mollayeva T (2016). The Pittsburgh sleep quality index as a screening tool for sleep dysfunction in clinical and non-clinical samples: A systematic review and meta-analysis. Sleep. Med Rev..

[34] Cook KF (2013). Pain assessment using the NIH Toolbox. Neurology.

[35] Alschuler KN, Jensen MP, Ehde DM (2012). Defining mild, moderate, and severe pain in persons with multiple sclerosis. Pain. Med..

[36] Zelman DC, Hoffman DL, Seifeldin R, Dukes EM (2003). Development of a metric for a day of manageable pain control: derivation of pain severity cut-points for low back pain and osteoarthritis. Pain.

[37] Cleeland CS (1984). The impact of pain on the patient with cancer. Cancer.

[38] Van Essen DC (2013). The WU-Minn human connectome project: an overview. Neuroimage.

[39] Shen X, Tokoglu F, Papademetris X, Constable RT (2013). Groupwise whole-brain parcellation from resting-state fMRI data for network node identification. Neuroimage.

[40] Backhaus J, Junghanns K, Broocks A, Riemann D, Hohagen F (2002). Test-retest reliability and validity of the Pittsburgh Sleep Quality Index in primary insomnia. J. Psychosom. Res.

[41] Dietch JR (2016). Psychometric evaluation of the PSQI in U.S. College students. J. Clin. Sleep. Med..

[42] Aloba OO, Adewuya AO, Ola BA, Mapayi BM (2007). Validity of the Pittsburgh Sleep Quality Index (PSQI) among Nigerian university students. Sleep. Med..

[43] Bulik-Sullivan B (2015). An atlas of genetic correlations across human diseases and traits. Nat. Genet..

[44] Choi SW, O’Reilly PF (2019). PRSice-2: Polygenic Risk Score software for biobank-scale data. GigaScience.

[45] Hayes, A. F. *Introduction to Mediation, Moderation, and Conditional Process Analysis A Regression-Based Approach* (Guilford Press, 2013).

[46] Bowden J, Davey Smith G, Burgess S (2015). Mendelian randomization with invalid instruments: effect estimation and bias detection through Egger regression. Int J. Epidemiol..

[47] Bowden J, Davey Smith G, Haycock PC, Burgess S (2016). Consistent estimation in Mendelian randomization with some invalid instruments using a weighted median estimator. Genet. Epidemiol..

[48] Verbanck M, Chen CY, Neale B, Do R (2018). Detection of widespread horizontal pleiotropy in causal relationships inferred from Mendelian randomization between complex traits and diseases. Nat. Genet..

[49] Qi G, Chatterjee N (2019). Mendelian randomization analysis using mixture models for robust and efficient estimation of causal effects. Nat. Commun..

[50] Godfrey KM (2016). Familial contributions to self-reported sleep and pain in female twins. Pain. Med..

[51] Ong WY, Stohler CS, Herr DR (2019). Role of the prefrontal cortex in pain processing. Mol. Neurobiol..

[52] Yaffe K, Falvey CM, Hoang T (2014). Connections between sleep and cognition in older adults. Lancet Neurol..

[53] Baliki MN, Geha PY, Apkarian AV, Chialvo DR (2008). Beyond feeling: chronic pain hurts the brain, disrupting the default-mode network dynamics. J. Neurosci..

[54] Clausen AN (2017). PTSD and cognitive symptoms relate to inhibition-related prefrontal activation and functional connectivity. Depress Anxiety.

[55] Preusser S (2015). The perception of touch and the ventral somatosensory pathway. Brain.

[56] van Dalfsen JH, Markus CR (2018). The influence of sleep on human hypothalamic-pituitary-adrenal (HPA) axis reactivity: a systematic review. Sleep. Med. Rev..

[57] Yang, S. & Chang, M. C. Chronic pain: structural and functional changes in brain structures and associated negative affective states. *Int. J. Mol. Sci*. **20**, 3130 (2019). 10.3390/ijms20133130 PMC6650904 31248061

[58] Apkarian AV (2016). Role of adult hippocampal neurogenesis in persistent pain. Pain.

[59] Mutso AA (2014). Reorganization of hippocampal functional connectivity with transition to chronic back pain. J. Neurophysiol..

[60] Moscovitch M, Cabeza R, Winocur G, Nadel L (2016). Episodic memory and beyond: the hippocampus and neocortex in transformation. Annu. Rev. Psychol..

[61] Milad MR (2007). Recall of fear extinction in humans activates the ventromedial prefrontal cortex and hippocampus in concert. Biol. Psychiatry.

[62] Hamaker EL, Kuiper RM, Grasman RP (2015). A critique of the cross-lagged panel model. Psychol. Methods.

[63] Finnerup NB (2015). Pharmacotherapy for neuropathic pain in adults: a systematic review and meta-analysis. Lancet Neurol..

[64] Riemann D (2015). The neurobiology, investigation, and treatment of chronic insomnia. Lancet Neurol..

[65] Pieretti S (2016). Gender differences in pain and its relief. Ann. Ist. Super. Sanita.

[66] Theorell-Haglow J (2018). Gender differences in obstructive sleep apnoea, insomnia and restless legs syndrome in adults - what do we know? A clinical update. Sleep. Med. Rev..

