# Supplementary material for: Polygenic evidence and overlapped brain functional connectivities for the association between chronic pain and sleep disturbance
Source: Transl Psychiatry. 2020 Jul 24;10:252. doi: 10.1038/s41398-020-00941-z (PMC7381677; doi:10.1038/s41398-020-00941-z)
Supplement: Supplementary file 1 — Supplementary Information [file 41398_2020_941_MOESM1_ESM.docx]

**Supplementary Materials**

**Polygenic evidence and overlapped brain functional connectivities for the association between chronic pain and sleep disturbance**

Jie Sun^1,2,3^, Wei Yan^2^, Xing-Nan Zhang^2^, Xiao Lin^2^, Hui Li^2^, Yi-Miao Gong^2^, Xi-Mei Zhu^2^, Yong-Bo Zheng^2^, Xiang-Yang Guo^3^, Yun-Dong Ma^2^, Zeng-Yi Liu^2^, Lin Liu^2^, Jia-Hong Gao^4^, Michael V. Vitiello^5^, Su-Hua Chang^2,6,*^, Xiao-Guang Liu^1,7,*^, Lin Lu^2,6,*^

^1^ *Center for Pain Medicine, Peking University Third Hospital, Beijing 100191, China.*

^2^ *Peking University Sixth Hospital, Peking University Institute of Mental Health, NHC Key Laboratory of Mental Health (Peking University), National Clinical Research Center for Mental Disorders (Peking University Sixth Hospital),* *Beijing 100191, China.*

^3^*Department of Anesthesiology, Peking University Third Hospital, Beijing 100191, China.*

^4^ *Center for MRI Research, Peking University, Beijing 100871, China.*

^5^*Department of Psychiatry and Behavioral Sciences, University of Washington, Seattle WA, 98195, USA*

^6^ *Research Unit of Diagnosis and Treatment of Mood Cognitive Disorder (2018RU006), Chinese Academy of Medical Sciences, Beijing 100191, China.*

^7^*Department of Orthopedics, Peking University Third Hospital, Beijing 100191, China.*

^*^ **Correspondence**: Suhua Chang (changsh@bjmu.edu.cn), Lin Lu (linlu@bjmu.edu.cn), or Xiaoguang Liu (xglius@vip.sina.com).

**Supplementary Methods**

**Phenotypes**

The sleep phenotype of participants in the HCP was assessed by Pittsburgh Sleep Quality Index (PSQI), a validated self-reported assessment of sleep disturbance^1^. The PSQI consists of a total score, which sums 7 component scores: subjective sleep quality, sleep latency, sleep duration, habitual sleep efficiency, sleep disturbances, use of sleeping medication, and daytime dysfunction in the past month. Participants’ self-reported experience of pain was measured by the NIH Toolbox Pain Intensity Survey^2^. Pain Intensity Survey consists of one self-report item asking “In the past 7 days, how would you rate your pain on average?” is scored on a numerical rating scale from 0 to 10, in which 0 denotes no pain and 10 denotes worst imaginable pain. The phenotype distributions of the two phenotypes are shown in Supplementary Figure 1. Since the distribution of pain intensity score was not normal, we used a binary variable for pain (chronic pain binary, CPb). According to previous studies^3^, a pain intensity score≥6 denotes severe pain, which was set as 1, and a pain intensity score ≤ 5 was set as 0.

**Genotype data quality control**

The genotype data was requested from dbGaP under access number phs001364. Among the total 1206 participants, 1142 subjects were genotyped using Infinium Multi-Ethnic Genotyping Array. The SNPs with call rates < 95% (45,987), minor allele frequency (MAF) < 0.01 (842,872) were removed. Samples from individuals with call rate < 95% (1 individuals), autosomal heterozygosity > 5 s.d. away from the mean (0 individual) or being one of a pair of individuals with proportion identity by descent (IBD) PI_HAT ≥ 0.2 were removed (since HCP contained many twins and siblings, 703 individuals were removed by this condition; the one with lower call rate was removed). Principal component analysis (PCA) was then performed for all samples. A total of 42,119 genotyped autosomal SNPs, which were in low linkage disequilibrium (LD) (MAF > 0.05 and r^2^ < 0.05 for each pair of SNPs) and absent from the 5 long-range LD regions^4^, were included in the PCA using EIGENSOFT 4.2 software^5,6^. 41 outliers were identified by PCA, which were further removed from the samples. Quality control (--geno 0.05, --mind 0.05, --maf 0.01, --hwe 0.000001) were conducted again and the final dataset included 429 individuals with 1,169,182 SNPs for polygenic score association analysis. The PCA plots for the top 10 principal components (PCs) are shown in Supplementary Figure 2. The top 10 PCs with Tracy-Widom test p-value<0.05 were used as covariates in subsequent association analysis.

**Imaging data and construction of whole-brain functional network**

Parameter of scanning is TR = 720 ms, TE = 33.1 ms, 72 slices, 2.0 mm isotropic resolution, 52 degree flip angle, multiband factor = 8. Preprocessing was conducted by HCP using the HCP pipeline^7^. The participants with four resting-state data (two scans, oblique axial acquisitions alternated between phase encoding in a right-to-left direction in one run and phase encoding in a left-to-right direction in the other run within each scan) were used for the construction of whole-brain functional network.

After preprocessing, the gray matter of the whole brain was parcellated into 250 regions of interest employing the Shen atlas^8^, which has been validated in resting-state functional magnetic resonance imaging (fMRI) studies^9,10^. Nodal signals were created by averaging the regional blood oxygen level-dependent signals of all voxels within each region. Pearson cross correlations between all pairwise combinations of the region signals were calculated for each participant, followed by z transformation to improve normality. The whole-brain functional connectivity network (250 × 250 regions with 31,125 edges) was constructed by further averaging the correlation coefficients of two directions of two scans.

**LD score regression and polygenic score association analyses for the association between chronic pain and sleep disturbance**

LD score regression^11,12^ was analyzed using the python script and European LD scores downloaded from https://github.com/bulik/ldsc. PGS association analysis was conducted by using PRSice v2^13^. Before creating the scores, clumping was used to obtain SNPs in linkage equilibrium with an r^2^<0.1 within a 250 bp window. The PGS at five p-value thresholds (5.0 ×10^-5^, 5.0 ×10^-4^, 5.0 ×10^-3^, 0.05, 0.5) were extracted. The association of the PGS with phenotype was analyzed using regression models (linear regression for PSQI score, logistic regression for CPb) with same covariates above, and 10 PCs from the PCA of the genotype data. The results with p-value < 0.01 were considered to be significant.

**Supplementary Table 1**. Polygenic score (PGS) association analysis results for the association of PGS of chronic pain with sleep disturbance and vice versa. PSQI is the Pittsburgh sleep quality index, CPb is the binary chronic pain variable.

| **Discovery** | **Target** | **Threshold** | **PRS.R2** | **Coefficient** | **SE** | **p-value** | **Num SNP** | **P.adjust** |
| --- | --- | --- | --- | --- | --- | --- | --- | --- |
| Chronic pain | PSQI total score | 5.00×10^-5^ | 0.0159 | 1051.62 | 384.29 | 6.47×10^-3^ | 513 | **0.0324** |
|  |  | 5.00×10^-4^ | 0.0033 | 983.34 | 790.33 | 0.2141 | 1573 | 1.0000 |
|  |  | 5.00×10^-3^ | 0.0033 | 2031.46 | 1635.40 | 0.2149 | 5658 | 1.0000 |
|  |  | 0.05 | 0.0026 | 4439.49 | 4030.65 | 0.2713 | 23777 | 1.0000 |
|  |  | 0.5 | 3.38×10^-6^ | -484.65 | 12278.00 | 0.9685 | 105272 | 1.0000 |
| Sleep disturbance | CPb | 5.00×10^-5^ | 3.53×10^-4^ | 53.45 | 238.32 | 0.8226 | 195 | 1.0000 |
|  |  | 5.00×10^-4^ | 0.0269 | 604.45 | 303.87 | 0.0467 | 790 | 0.2334 |
|  |  | 5.00×10^-3^ | 0.0174 | 1264.66 | 831.78 | 0.1284 | 3911 | 0.6420 |
|  |  | 0.05 | 0.0138 | 2466.28 | 1836.62 | 0.1793 | 20622 | 0.8966 |
|  |  | 0.5 | 0.1102 | 24399.30 | 7382.47 | 9.50×10^-4^ | 109519 | **4.75×10^-3^** |

**Supplementary Table 2**. Mediation analysis results for the nine shared functional connectivities. Path *a* is the effect of X on M, path *b* is the effect of M on Y, path *c* is the effect of X on Y (total effect), *c’* is the indirect effect of X on Y. PSQI is the Pittsburgh sleep quality index, CPb is the binary chronic pain variable.

| **M** | | **X** | **Y** | **path a: X->M** | | | **path b: M->Y** | | | **path c: X->Y** | | | | **path c': X->Y indirect** | | |
| --- | --- | --- | --- | --- | --- | --- | --- | --- | --- | --- | --- | --- | --- | --- | --- | --- |
| **Brain Region1** | **Brain Region2** |  |  | **Beta** | **SE** | **p-value** | **Beta** | **SE** | **p-value** | **Beta** | **SE** | **p-value** | **Beta** | | **SE** | **p-value** |
| Right middle temporal gyrus | Right hippocampus | PSQI | CPb | 0.0081 | 0.0022 | 0.0002 | 3.1901 | 1.0123 | 0.0016 | 0.2195 | 0.0529 | <10^-4^ | 0.0259 | | 0.011 | **0.0186** |
|  |  | CPb | PSQI | 0.1097 | 0.0308 | 0.0004 | 1.4482 | 0.4583 | 0.0016 | 2.2516 | 0.4443 | <10^-4^ | 0.1588 | | 0.0723 | **0.0208** |
| Right precentral gyrus | Left fusiform gyrus | PSQI | CPb | 0.0083 | 0.0026 | 0.0012 | 2.6867 | 0.8573 | 0.0019 | 0.2134 | 0.0538 | 0.0001 | 0.0223 | | 0.0101 | **0.0278** |
|  |  | CPb | PSQI | 0.131 | 0.036 | 0.0003 | 1.0505 | 0.3936 | 0 | 2.2729 | 0.4451 | <10^-4^ | 0.1376 | | 0.0655 | **0.0355** |
| Right precentral gyrus | Left hippocampus | PSQI | CPb | 0.0077 | 0.0026 | 0.0034 | 2.5991 | 0.813 | 0.0014 | 0.2209 | 0.0524 | <10^-4^ | 0.0199 | | 0.0095 | **0.0351** |
|  |  | CPb | PSQI | 0.1364 | 0.0367 | 0.0002 | 0.906 | 0.3864 | 0.0192 | 2.2869 | 0.4456 | <10^-4^ | 0.1236 | | 0.0639 | 0.0531 |
| Right precentral gyrus | Left inferior temporal gyrus | PSQI | CPb | 0.0107 | 0.0026 | 0.0001 | 2.1637 | 0.7989 | 0.0068 | 0.2188 | 0.0534 | <10^-4^ | 0.0231 | | 0.0105 | **0.0275** |
|  |  | CPb | PSQI | 0.1178 | 0.0371 | 0.0016 | 1.3537 | 0.3799 | 0.0004 | 2.251 | 0.4431 | <10^-4^ | 0.1595 | | 0.0688 | **0.0204** |
| Right inferior frontal gyrus, triangular part | Left temporal pole: middle temporal gyrus | PSQI | CPb | 0.0089 | 0.0028 | 0.0017 | 2.4176 | 0.792 | 0.0023 | 0.2264 | 0.053 | <10^-4^ | 0.0215 | | 0.0101 | **0.0326** |
|  |  | CPb | PSQI | 0.1414 | 0.0396 | 0.0004 | 0.9243 | 0.3573 | 0.0098 | 2.2798 | 0.4451 | <10^-4^ | 0.1307 | | 0.064 | **0.0411** |
| Right postcentral gyrus | Left middle frontal gyrus | PSQI | CPb | 0.009 | 0.0026 | 0.0006 | 2.2756 | 0.8188 | 0.0054 | 0.2174 | 0.053 | <10^-4^ | 0.0205 | | 0.0097 | **0.0348** |
|  |  | CPb | PSQI | 0.1158 | 0.0367 | 0.0017 | 1.14 | 0.3853 | 0.0032 | 2.2785 | 0.444 | <10^-4^ | 0.132 | | 0.0628 | **0.0355** |
| Right precentral gyrus | Left temporal pole: middle temporal gyrus | PSQI | CPb | 0.0093 | 0.0026 | 0.0003 | 2.1786 | 0.8112 | 0.0072 | 0.2174 | 0.0523 | <10^-4^ | 0.0203 | | 0.0097 | **0.0354** |
|  |  | CPb | PSQI | 0.1176 | 0.0364 | 0.0013 | 1.2094 | 0.3885 | 0.0019 | 2.2683 | 0.4439 | <10^-4^ | 0.1422 | | 0.065 | **0.0286** |
| Left superior frontal gyrus, dorsolateral | Left anterior cingulate & paracingulate gyri | PSQI | CPb | 0.0092 | 0.003 | 0.0021 | 2.1693 | 0.7794 | 0.0054 | 0.2241 | 0.0528 | <10^-4^ | 0.0199 | | 0.0099 | **0.0445** |
|  |  | CPb | PSQI | 0.1325 | 0.0418 | 0.0016 | 0.8762 | 0.3385 | 0.0098 | 2.2944 | 0.4445 | <10^-4^ | 0.1161 | | 0.0596 | 0.0515 |
| Right precentral gyrus | Left superior frontal gyrus, dorsolateral | PSQI | CPb | 0.0104 | 0.0029 | 0.0004 | 2.0233 | 0.7457 | 0.0067 | 0.2229 | 0.0529 | <10^-4^ | 0.021 | | 0.01 | **0.0355** |
|  |  | CPb | PSQI | 0.1253 | 0.0413 | 0.0025 | 1.054 | 0.3424 | 0.0021 | 2.2785 | 0.4437 | <10^-4^ | 0.132 | | 0.0627 | **0.0353** |

**Supplementary Table 3**. PGS association analysis results for the association of polygenic score of chronic pain/sleep disturbance with their shared brain functional connectivity.

| **Discovery** | **Target functional connectivity** | | **Threshold** | **R^2^** | **Beta** | **SE** | **p-value** | **No. SNP** | **P_adjust** |
| --- | --- | --- | --- | --- | --- | --- | --- | --- | --- |
|  | **Brain Region 1** | **Brain Region 2** |  |  |  |  |  |  |  |
| Chronic pain | Right middle temporal gyrus | Right hippocampus | 0.0016 | 0.0219 | 223.823 | 76.902 | **0.0038** | 2917 | **0.0402** |
| Chronic pain | Right precentral gyrus | Left fusiform gyrus | 0.00015 | 0.0122 | -94.291 | 42.368 | **0.0267** | 864 | 0.327 |
| Chronic pain | Right precentral gyrus | Left hippocampus | 0.0043 | 0.0031 | 141.132 | 128.59 | 0.2731 | 5144 | 0.9962 |
| Chronic pain | Right precentral gyrus | Left inferior temporal gyrus | 0.014 | 0.0036 | 238.004 | 203.916 | 0.2439 | 10731 | 0.9977 |
| Chronic pain | Right inferior frontal gyrus, triangular part | Left temporal pole: middle temporal gyrus | 0.032 | 0.0046 | 376.934 | 285.34 | 0.1873 | 18023 | 0.9955 |
| Chronic pain | Right postcentral gyrus | Left middle frontal gyrus | 0.026 | 0.0052 | 352.975 | 250.451 | 0.1596 | 15857 | 0.9955 |
| Chronic pain | Right precentral gyrus | Left temporal pole: middle temporal gyrus | 0.0001 | 0.0076 | -65.264 | 38.466 | 0.0906 | 719 | 0.9628 |
| Chronic pain | Left superior frontal gyrus, dorsolateral | Left anterior cingulate & paracingulate gyri | 0.005 | 0.0042 | 184.99 | 147.833 | 0.2116 | 5658 | 0.9999 |
| Chronic pain | Right precentral gyrus | Left superior frontal gyrus, dorsolateral | 0.00065 | 0.0094 | 160.701 | 83.107 | 0.0539 | 1802 | 0.9267 |
| Sleep disturbance | Right middle temporal gyrus | Right hippocampus | 1 | 0.0177 | -991.947 | 379.878 | **0.0094** | 163621 | 0.1507 |
| Sleep disturbance | Right precentral gyrus | Left fusiform gyrus | 0.02285 | 0.0084 | -139.238 | 75.204 | 0.0649 | 11542 | 0.8201 |
| Sleep disturbance | Right precentral gyrus | Left hippocampus | 0.03505 | 0.0041 | -122.43 | 97.068 | 0.208 | 15886 | 0.9988 |
| Sleep disturbance | Right precentral gyrus | Left inferior temporal gyrus | 0.4811 | 0.0140 | 703.664 | 303.995 | **0.0212** | 106770 | 0.658 |
| Sleep disturbance | Right inferior frontal gyrus, triangular part | Left temporal pole: middle temporal gyrus | 0.0008 | 0.0196 | 60.572 | 22.096 | **0.0064** | 1075 | 0.3957 |
| Sleep disturbance | Right postcentral gyrus | Left middle frontal gyrus | 0.007 | 0.0159 | 125.321 | 50.215 | **0.013** | 4918 | 0.6523 |
| Sleep disturbance | Right precentral gyrus | Left temporal pole: middle temporal gyrus | 5.00E-05 | 0.0155 | 24.062 | 9.863 | **0.0152** | 195 | 0.7266 |
| Sleep disturbance | Left superior frontal gyrus, dorsolateral | Left anterior cingulate & paracingulate gyri | 5.00E-05 | 0.0041 | -13.363 | 10.835 | 0.2183 | 195 | 1 |
| Sleep disturbance | Right precentral gyrus | Left superior frontal gyrus, dorsolateral | 0.00485 | 0.0131 | 120.659 | 52.59 | **0.0224** | 3843 | 0.903 |

**Supplementary Table 4**. Mediation analysis result for the indirect effect of polygenic score of chronic pain/sleep disturbance on PSQI/CPb through the mediation of functional connectivity (FC) between right middle temporal gyrus and right hippocampus. PSQI is the Pittsburgh sleep quality index, CPb is the binary chronic pain variable.

| **M** | **X** | **Y** | **Path** | **Beta** | **SE** | **P-value** |
| --- | --- | --- | --- | --- | --- | --- |
| FC: right middle temporal gyrus – right hippocampus | sleep disturbance PGS associated with the FC | CPb | path a: X->M | -559.0037 | 202.7029 | **0.0061** |
|  |  |  | path b: M->Y | 3.5269 | 1.7845 | **0.0481** |
|  |  |  | path c: X->Y | -8570.1655 | 7316.0438 | 0.2414 |
|  |  |  | path c': X->Y indirect | -1971.543 | 1279.4415 | 0.1233 |
|  | chronic pain PGS associated with the FC | PSQI | path a: X->M | 236.8976 | 75.1083 | **0.0018** |
|  |  |  | path b: M->Y | 2.1928 | 0.8106 | **0.0072** |
|  |  |  | path c: X->Y | 84.1543 | 1145.4179 | 0.9415 |
|  |  |  | path c': X->Y indirect | 519.4613 | 260.2043 | **0.0459** |

**Supplementary Figure 1.** Phenotype distribution for PSQI total score and pain intensity score.

**
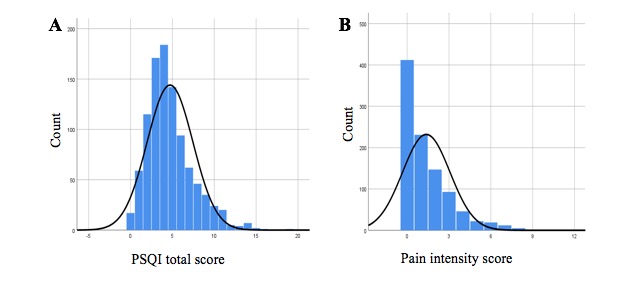
**

**Supplementary Figure 2**. Plot for the population stratification using the 10 principal components from the principal component analysis (PCA) for the genome-wide genotype data of HCP.


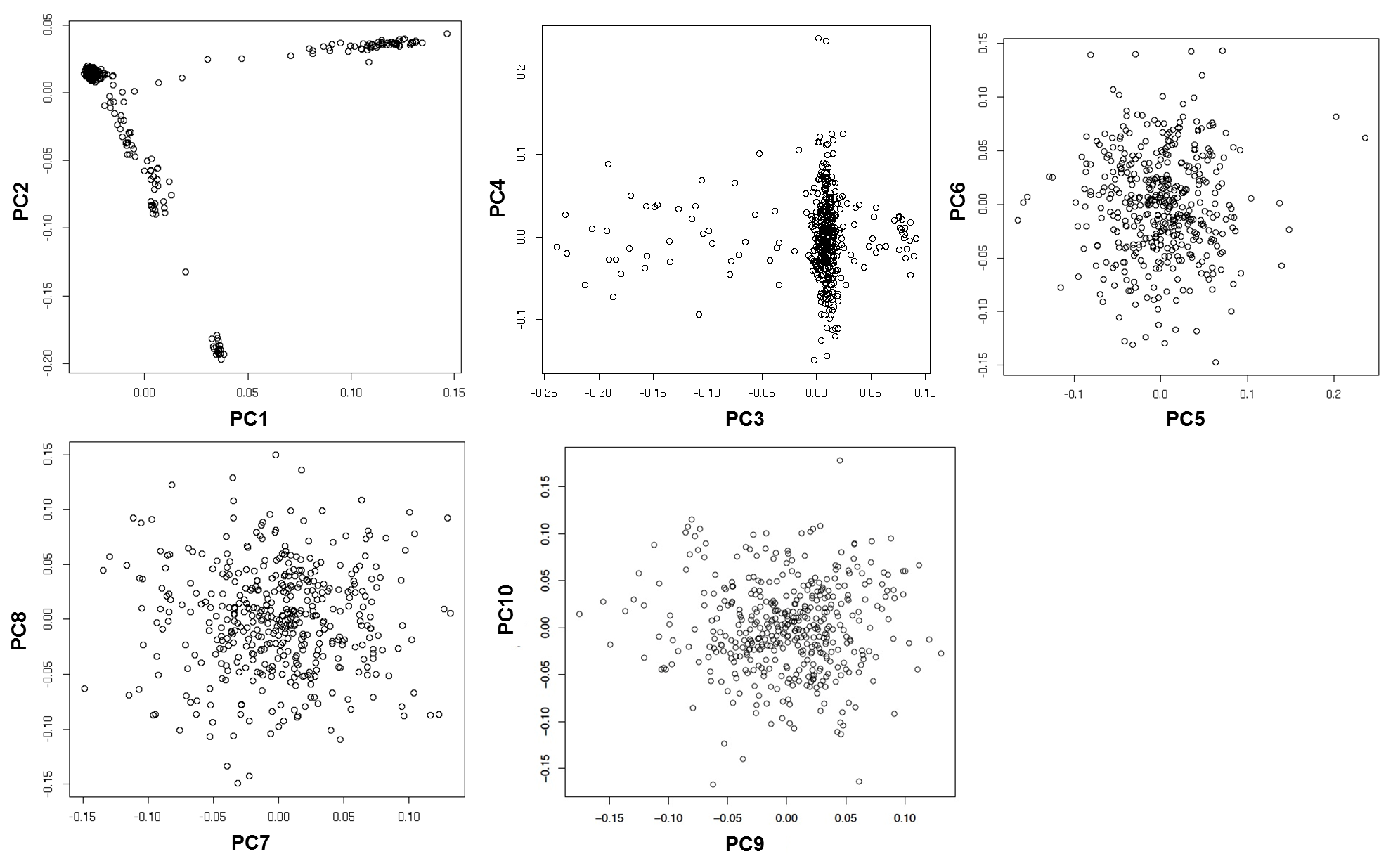


**References**

1 Mollayeva, T. *et al.* The Pittsburgh sleep quality index as a screening tool for sleep dysfunction in clinical and non-clinical samples: A systematic review and meta-analysis. *Sleep Med Rev* **25**, 52-73 (2016).

2 Cook, K. F. *et al.* Pain assessment using the NIH Toolbox. *Neurology* **80**, S49-53 (2013).

3 Alschuler, K. N., Jensen, M. P. & Ehde, D. M. Defining mild, moderate, and severe pain in persons with multiple sclerosis. *Pain Med* **13**, 1358-1365 (2012).

4 Chen, J. *et al.* Genetic structure of the Han Chinese population revealed by genome-wide SNP variation. *Am.J.Hum.Genet.* **85**, 775-785 (2009).

5 Patterson, N., Price, A. L. & Reich, D. Population structure and eigenanalysis. *PLoS Genet* **2**, e190(2006).

6 Price, A. L. *et al.* Principal components analysis corrects for stratification in genome-wide association studies. *Nat.Genet.* **38**, 904-909 (2006).

7 Van Essen, D. C. *et al.* The WU-Minn Human Connectome Project: an overview. *Neuroimage* **80**, 62-79(2013).

8 Shen, X., Tokoglu, F., Papademetris, X. & Constable, R. T. Groupwise whole-brain parcellation from resting-state fMRI data for network node identification. *NeuroImage* **82**, 403-415(2013).

9 Rosenberg, M. D. *et al.* A neuromarker of sustained attention from whole-brain functional connectivity. *Nat.Neurosci.* **19**, 165-171, doi:10.1038/nn.4179 (2016).

10 Cheng, W., Rolls, E. T., Ruan, H. & Feng, J. Functional Connectivities in the Brain That Mediate the Association Between Depressive Problems and Sleep Quality. *JAMA psychiatry* **75**, 1052-1061(2018).

11 Bulik-Sullivan, B. K. *et al.* LD Score regression distinguishes confounding from polygenicity in genome-wide association studies. *Nat.Genet.* **47**, 291-295(2015).

12 Bulik-Sullivan, B. *et al.* An atlas of genetic correlations across human diseases and traits. *Nat.Genet.* **47**, 1236-1241(2015).

13 Choi, S. W. & O'Reilly, P. F. PRSice-2: Polygenic Risk Score software for biobank-scale data. *GigaScience* **8**,7(2019).
